# Efficient and Selective, In Vitro and In Vivo, Antimicrobial Photodynamic Therapy with a Dicationic Chlorin in Combination with KI

**DOI:** 10.1021/acsinfecdis.4c00492

**Published:** 2024-08-16

**Authors:** Anita S. Amorim, Zoe A. Arnaut, Ana I. Mata, Barbara Pucelik, Agata Barzowska, Gabriela J. da Silva, Mariette M. Pereira, Janusz M. Dąbrowski, Luis G. Arnaut

**Affiliations:** † CQC-IMS, Chemistry Department, University of Coimbra, Coimbra 3004-535, Portugal; ‡ Faculty of Chemistry, Jagiellonian University, Kraków 30-387, Poland; § Faculty of Pharmacy of the University of Coimbra and Center for Neurosciences and Cell Biology, Coimbra 3000-548, Portugal; ∥ Łukasiewicz Research Network – Kraków Institute of Technology, Kraków 30-418, Poland

**Keywords:** photodynamic inactivation, antimicrobial resistance, porphyrinoids, biofilms, infection

## Abstract

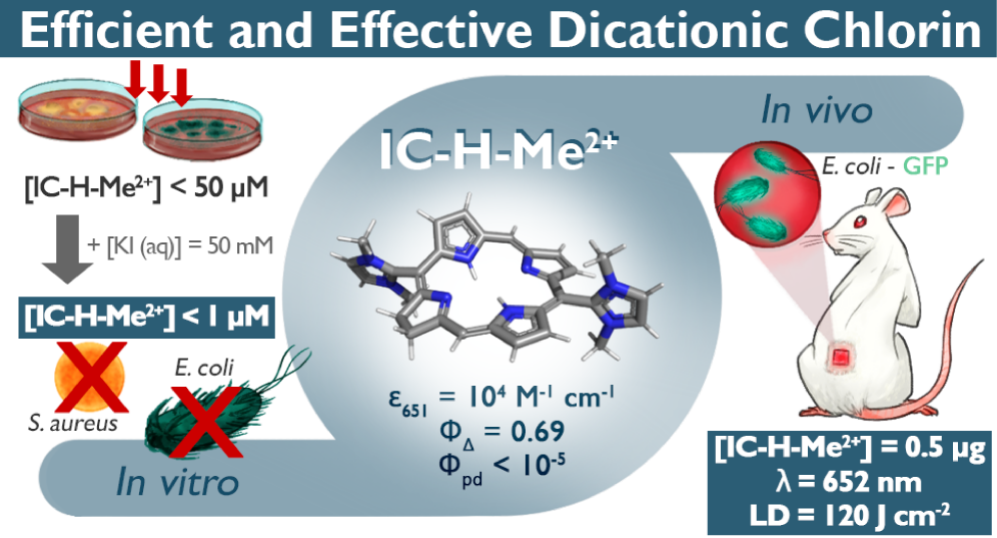

Various cationic photosensitizers employed in antimicrobial photodynamic therapy (aPDT) have the ability to photoinactivate planktonic bacteria under conditions of low phototoxicity to mammalian cells and without generating antimicrobial resistance (AMR). However, the photoinactivation of biofilms requires orders-of-magnitude higher photosensitizer concentrations, which become toxic to host cells. Remarkably, the bactericidal effect of a dicationic di-imidazolyl chlorin toward planktonic *S. aureus* and *E. coli* was observed in this work for concentrations below 400 nM under illumination at 660 nm and below 50 μM for the corresponding biofilms. At the latter concentrations, the chlorin is phototoxic toward human keratinocyte cells. However, in the presence of 50 mM KI, bactericidal concentrations are reduced to less than 50 nM for planktonic bacteria and to less than 1 μM for biofilms. It is shown that the potentiation with KI involves the triiodide anion. This potentiation elicits a bactericidal effect without appreciable cytotoxicity to keratinocytes. It becomes possible to selectively inactivate biofilms with aPDT. An exploratory study treating mice with wounds infected with *E. coli* expressing GFP with 20 μM chlorin and 120 J cm^–2^ at 652 nm confirmed the potential of this chlorin to control localized infections.

The combination of a dye and light to kill microorganisms was first reported by the laboratory of von Tappeiner in the early 1900s.^1,2^ Today, it is well established that in addition to the dye (i.e., a photosensitizer molecule) and light, molecular oxygen is also required to generate the cytotoxic species that inactivate microorganisms. In antimicrobial photodynamic therapy (aPDT), light is absorbed by a photosensitizer and used to generate singlet oxygen (^1^O_2_) via energy transfer, or to generate other reactive oxygen species (ROS) via electron transfer reactions.^3,4^ aPDT evolved into a mature field with a variety of clinical uses.^5^ Various cationic photosensitizers were shown to inactivate planktonic Gram-positive and Gram-negative bacteria,^6^ but azine photosensitizers such as acridine orange and methylene blue (MB), related to the original dyes investigated by von Tappeiner, are still widely used in antimicrobial PDT.^4^

The slow clinical translation of new photosensitizers for aPDT is related with at least 3 factors: (i) the reliance on antimicrobials to treat all kinds of infectious diseases; (ii) the poor predictive value of the photoinactivation of planktonic bacteria with respect to biofilms and animal models of infection; (iii) the lack of investment in clinical studies with new photosensitizers for aPDT. Indeed, the success of antibiotics from their discovery until the recognition that antimicrobial resistance (AMR) will become the leading public health threat of the 21st century,^7^ overshadowed other methods to treat infections. This drove the global use of antibiotics in humans, livestock and aquaculture to the alarming level of 100,000 tones per year.^8^ The rising AMR seems to be unstoppable and the ever increasing rates of resistance emergence to new antibiotics shows that alternative approaches to infectious diseases are desperately needed.^9^

The repeated exposure of microorganisms to sublethal aPDT protocols does not diminish their susceptibility to aPDT or their response to subsequent antimicrobial treatment.^10,11^ The recognition that aPDT is a valuable tool to overcome AMR gave a new impulse to the development of photosensitizers capable of inactivating bacteria in planktonic and biofilm forms, in conditions of low toxicity to mammalian cells. We found that phthalocyanines and porphyrins with positively charged imidazolyl groups can reduce colonies of bacteria by >5 log units at submicromolar concentrations with light doses that are not toxic to mammalian cells.^12−14^ Porphyrins performed better than phthalocyanines, and dicationic porphyrins were more effective toward biofilms than tetra-cationic porphyrins. The lowest molecular weight porphyrin of our series, IP-H-Me^2+^ (501 Da), shown in Scheme 1, was tested in aPDT of excision wounds infected with *E. coli*, and shown to significantly reduce the infection with one single treatment (25 μM and 120 J cm^–2^ at 420 nm). However, blue light is strongly absorbed and scattered by human tissues, and can only have superficial effects. We shifted our attention to the corresponding chlorin, IC-H-Me^2+^ in Scheme 1, and showed that it was remarkably effective in photoinactivating virus at submicromolar concentrations when combined with low light doses (<5 J cm^–2^ at 650 nm) and short incubation times (<2 h) that spare mammalian cells.^15^ The dicationic chlorin IC-H-Me^2+^ offers intense absorption in the red (ε_651_ = 10^4^ M^–1^ cm^–1^), high singlet oxygen quantum yield (Φ_Δ_ = 0.69), low photodecomposition quantum yield (Φ_pd_ < 10^–5^), small size (503 Da), solubility in biocompatible vehicles and low dark toxicity to mammalian cells (EC_50_ > 100 μM), which have been identified as the physical and photochemical properties of a “perfect” photosensitizer for aPDT.^16^

**Scheme 1:**
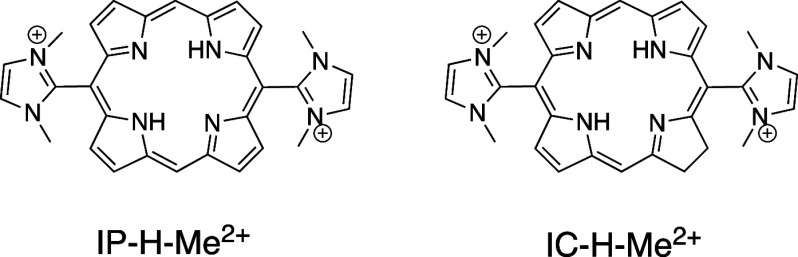
Molecular Structures of IP-H-Me^2+^ (5,15-bis(1,3-Dimethylimidazol-2-yl)porphyrinate) and IC-H-Me^2+^ (5,15-bis(1,3-Dimethylimidazol-2-yl)chlorinate)

In this work, we report aPDT against *S. aureus* (Gram-positive) and *E. coli* (Gram-negative) bacteria in planktonic and biofilm forms using IC-H-Me^2+^ and light at 650 nm. *E. coli* is the pathogen with the most deaths associated with AMR, followed by *S. aureus*.^7^ Together, *E. coli* and *S. aureus* infections account for 50% of the fatal burden attributed to AMR. Eradication of bacteria in biofilms requires antibiotic concentrations 100 to 1,000 times higher than for planktonic bacteria.^4,17,18^ In order to tackle this challenge, we explored combinations with potassium iodide, known to potentiate aPDT.^19−21^ Finally, we employed an animal model of infection to show that aPDT using 50 μL of a 20 μM chlorin solution with 120 J cm^–2^ at 652 nm controls the wound infection.

## Results and Discussion

**Figure 1:**
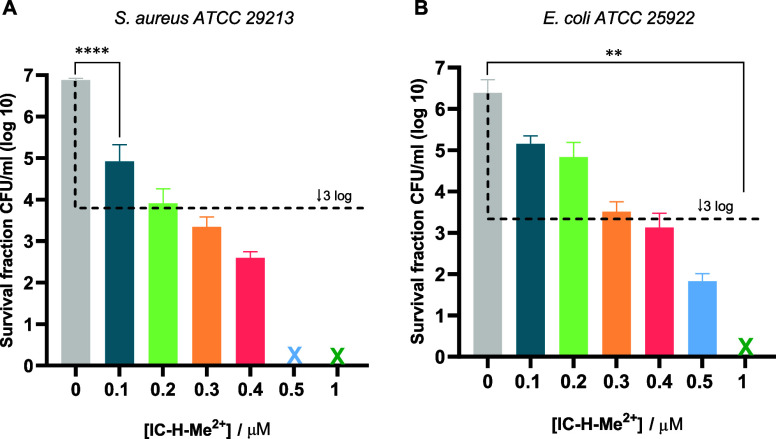
Photodynamic inactivation of planktonic *S. aureus* and *E. coli* using a light dose of 5 J cm^–2^, 1 h incubation time and the following of IC-H-Me^2+^ concentrations: 0 μM—gray, 0.1 μM—dark blue, 0.2 μM—light green, 0.3 μM—orange, 0.4 μM—red, 0.5 μM—light blue, 1 μM—dark green. A) *S. aureusATCC* 29213. B) *E. coliATCC* 25922. The dashed black line shows viability values for 99.9% (3 log units) inactivation of microorganisms and the cross, an inactivation to below the detection limit. The data expressed as mean value (*n* = 3) ± sem. For statistical analysis, one-way ANOVA was used (**p < 0.01, **** *p* < 0.0001).

The synthesis and characterization of 5,15-bis(1,3-dimethylimidazol-2-yl)chlorinate diiodide (IC-H-Me^2+^) was reported recently, including HPLC purity above 95%.^15^ We confirmed that this chlorin does not have significant cytotoxicity in the absence of light in the tens of micromolar range and that, under 5 J cm^–2^ at 650 nm after 1 h of incubation, cell viability only decreases to less than 80% when IC-H-Me^2+^ concentration is increased above 1 μM (Figure S1). In contrast, Figure 1 shows that, for 1 h incubation and 5 J cm^–2^, *S. aureus* colony-forming units per milliliter (CFU/ml) are reduced by >7 log units and *E. coli* by 5 log CFU/ml at 0.5 μM IC-H-Me^2+^. To place this value in perspective, it is useful to recall that a 2 log CFU/ml reduction of *E. coli* with 5 J cm^–2^ required 20 μM MB.^19^ Although the incubation time of MB was only 15 min, it is clear that our dicationic chlorin is several orders of magnitude more potent than MB. The closest phototoxicity reported for red/infrared absorbing photosensitizers is a dicationic bacteriochlorin reported by Lindsey and Hamblin, with 6 log CFU/ml inactivation of *E. coli* at 1 μM and 10 J cm^–2^.^22^ Here, we consider that a reduction by more than 3 log CFU/ml is a bactericidal effect.^4,23^

**Figure 2:**
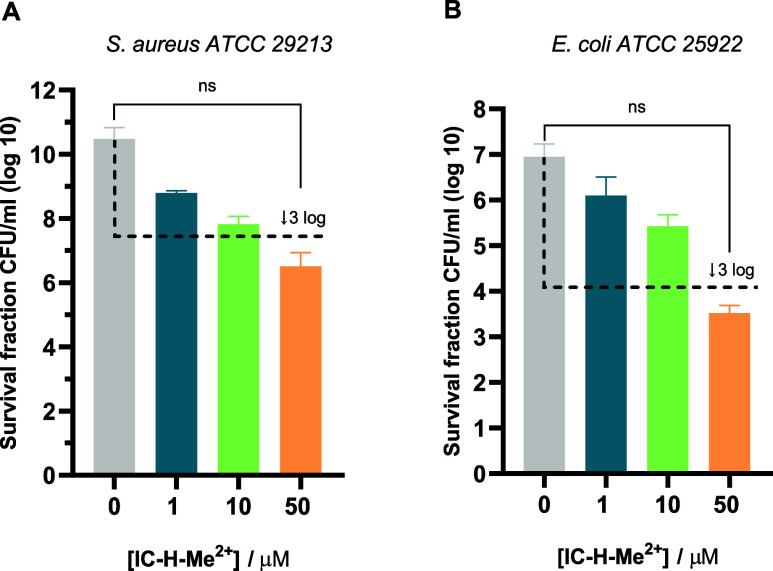
Photodynamic inactivation of *S. aureus* and *E. coli* biofilms with 5 J cm^–2^, after 1 h of incubation with IC-H-Me^2+^ at the following concentrations: 0 μM—gray, 1 μM—blue, 10 μM—green, 50 μM—orange. A) *S. aureusATCC* 29213 biofilms. B) *E. coliATCC 25922* biofilms. The dashed black line shows viability values for 99.9% (3 log units) inactivation of microorganisms. The data expressed as mean value (*n* = 3) ± sem. For statistical analysis, one-way ANOVA was used (ns, not significant).

As expected, a bactericidal effect in biofilms with 5 J cm^–2^ and 1 h of incubation required a chlorin concentration 100 times higher, i.e., 50 μM IC-H-Me^2+^ (Figure 2), than for planktonic bacteria. For comparison, Nonell and coworkers obtained a ∼1.5 log CFU/ml decrease in *E. coli* biofilms with 78 μM MB and 18 J cm^–2^.^24^ The only red-absorbing photosensitizer with aPDT potency comparable to that of IC-H-Me^2+^ is a MB-polymyxin conjugate, which achieved a 8 log CFU/ml reduction of *E. coli* biofilms with 50 μM and a light dose of 288 J cm^–2^.^4^ However, this conjugate has no effect against *S. aureus*.^25^ Photoinactivation of mature biofilms requires photosensitizer concentrations in the tens of micromolar range, which may become toxic to mammalian cells in the presence of light.^26^ The susceptibility of biofilms depends on the biofilm age and the more mature biofilms tend to have a poorer response to treatment. In our case, all the biofilms were grown for 24 h before incubation with the photosensitizer, which is slightly longer than in comparable studies.^24^ Potentiation of aPDT with nontoxic additives enables bactericidal effects at lower photosensitizer doses.^14,27^

**Figure 3:**
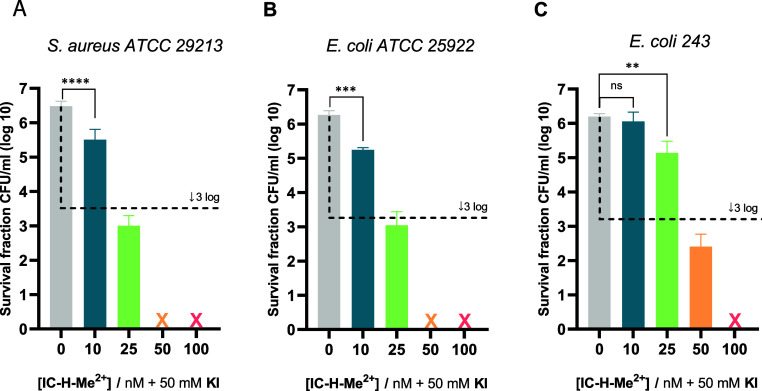
Photodynamic inactivation of planktonic bacteria with 50 mM KI, 5 J cm^–2^ and 1 h of incubation with the following IC-H-Me^2+^ concentrations: 0 nM—gray, 10 nM—blue, 25 nM—green, 50 nM—orange, 100 nM—red. A) *S. aureusATCC* 29213. B) *E. coliATCC 25922*. C) *E. coli243*. The dashed black line shows viability values for 99.9% (3 log units) inactivation of microorganisms and the cross represents inactivation to below the detection limit. The data expressed as mean value (*n* = 3) ± sem. For statistical analysis, one-way ANOVA was used (ns, not significant, **p < 0.01, and ***p < 0.001, **** *p* < 0.0001).

**Figure 4:**
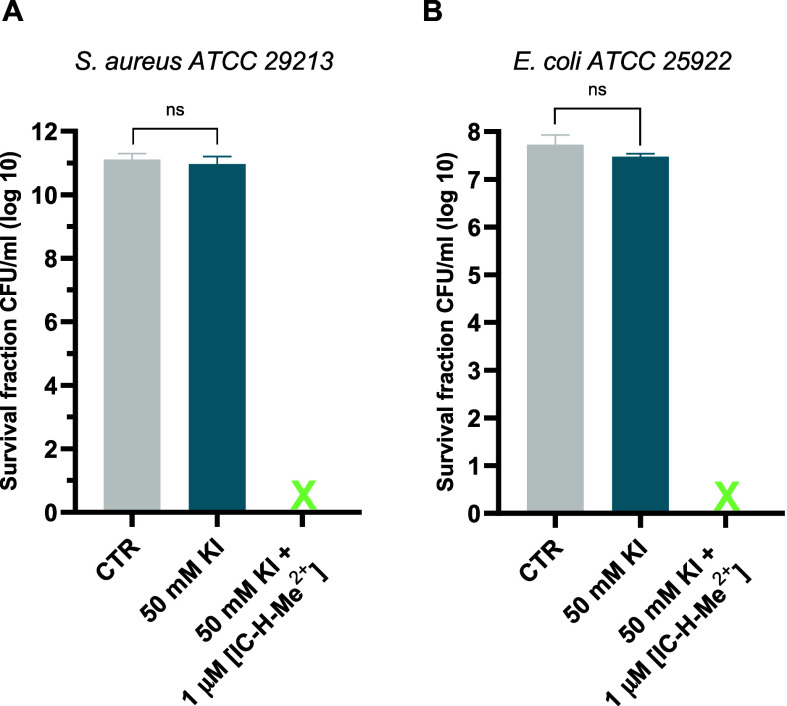
Photodynamic inactivation of biofilms with 50 mM KI, 5 J cm^–2^ and 1 h of incubation with 1 μM IC-H-Me^2+^; CTR, in gray, is a control (no KI, no photosensitizer, no light); blue refers to incubation with KI alone. A) *S. aureusATCC* 29213 biofilms. B) *E. coliATCC 25922* biofilms. The crosses represents inactivation to below the detection limit. The data expressed as mean value (*n* = 3) ± sem. For statistical analysis, one-way ANOVA was used (ns, not significant).

Hamblin showed that phototoxicity against planktonic bacteria is strongly enhanced in combinations of aPDT with KI.^19,20^ Literature on the potentiation of aPDT with KI in biofilms is sparse,^28^ but Hamblin and Wang found that planktonic *E. faecalis* eradication in the presence of 100 mM KI required 0.4 μM MB and 6 J cm^–2^, whereas in biofilm it required 10 μM MB and 30 J cm^–2^.^29^Figure 3 shows that KI indeed has a dramatic synergistic effect to inactivate planktonic bacteria: in the presence of 50 mM KI, the concentration of IC-H-Me^2+^ can be lowered to 50 nM and still be bactericidal with 5 J cm^–2^ after 1 h of incubation. This is an order of magnitude reduction relative to the concentration of IC-H-Me^2+^ alone to achieve a comparable inactivation. Figure 4 shows that the same level of success can be obtained in the inactivation of biofilms. Eradication of the biofilms is achieved with 1 μM IC-H-Me^2+^ in the presence of 50 mM KI and 5 J cm^–2^, whereas in the absence of KI we needed 50 μM IC-H-Me^2+^ to obtain a bactericidal effect with the same incubation time and light dose. This success must be tempered by the phototoxicity to mammalian cells that the combination of aPDT with KI may elicit.

**Figure 5:**
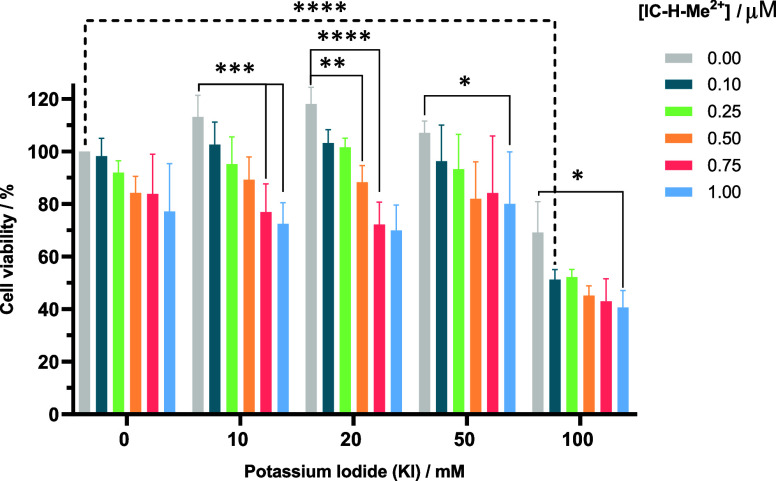
Photodynamic therapy of HaCaT cells using different concentrations of IC-H-Me^2+^ and KI, with 1 h of incubation and a light dose of 5 J cm^–2^. Differences between 0 μM IC-H-Me^2+^ + 0 mM KI and 0 μM IC-H-Me^2+^ + 100 mM KI are not statistically significant, but differences between 0 μM IC-H-Me^2+^ + 0 mM KI and groups with IC-H-Me^2+^ + 100 mM KI + light are statistically significant. The data expressed as mean value (*n* = 3) ± sem. For statistical analysis, one-way ANOVA was used (ns, not significant, *p < 0.05, **p < 0.01, and ***p < 0.001, **** *p* < 0.0001).

Although KI alone is not toxic to bacterial or eukaryotic cells at least up to a 50 mM concentration, and does not absorb visible light, the potentiation of phototoxicity observed in aPDT may also occur in photodynamically treated mammalian cells. This does not seem to have been reported in the literature. Cytotoxicity in the dark is presented in Figure S2. For 1 h of incubation, we only found cytotoxicity in the dark when the concentration of KI reaches 100 mM. Figure 5 shows the dependence of the viability of HaCaT cells with the increase in KI concentration for various chlorin concentrations and 5 J cm^–2^. Control experiments refer to cell viability in the absence of chlorin. Cell viability with 50 mM KI is higher than 70%, which we consider the upper limit of tolerable phototoxicity. Phototoxicity is significantly increased in the presence of 100 mM KI. At least when IC-H-Me^2+^ is illuminated with 5 J cm^–2^, KI should not exceed 50 mM, and incubation times should be less than 1 h, to prevent phototoxicity to the host cells. These were the conditions employed to eradicate biofilms in Figure 4.

**Figure 6:**
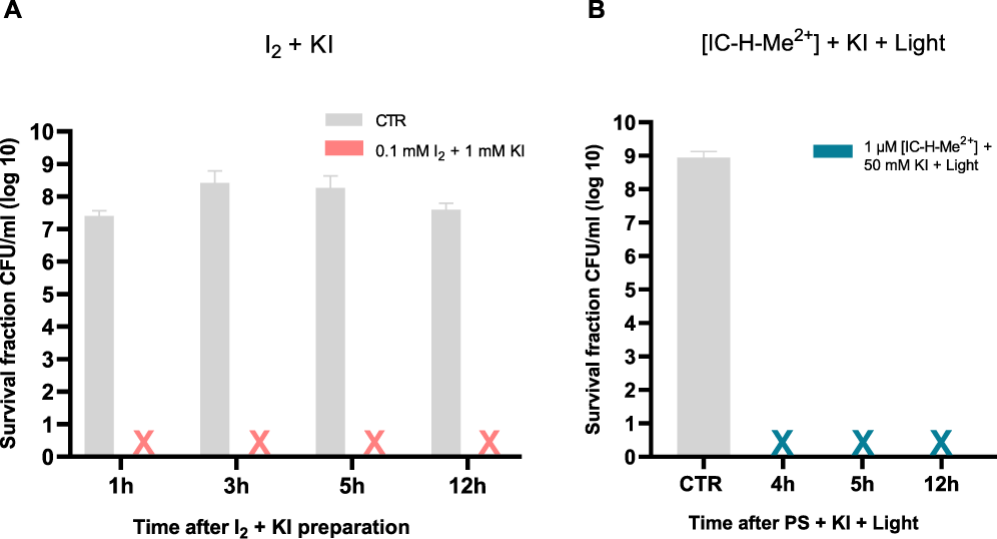
Survival of bacteria in *S. aureus* biofilms. A) Addition of 0.1 mM I_2_ to 1 mM KI solutions, followed 1 to 12 h later by the addition of the mixture to the biofilms. B) Illumination of 1 μM IC-H-Me^2+^ + 50 mM KI solutions with 5 J cm^–2^, and 4 to 12 h later, incubation of the solutions with the biofilms for 1 h in the dark. The crosses represents inactivation to below the detection limit. The data expressed as mean value (*n* = 3) ± sem.

The mechanism of aPDT potentiation by KI has been investigated in detail. It is believed that both short-lived reactive iodine species (e.g., I_2_^•–^)^19^ and/or long-lived molecular iodine (triiodide anion, I_3_^–^, in the presence of I^–^)^20,30^ may be involved.^29^ In order to identify the more relevant cytotoxic species in our systems, we investigated the inactivation of *S. aureus* biofilms in conditions where IC-H-Me^2+^ is illuminated in the presence of KI and added to the biofilms 4 to 12 h after the end of the illumination. Remarkably, Figure 6 shows that the bactericidal potential is not lost 12 h after the generation of cytotoxic species. This strongly suggests that I_3_^–^ mediates toxicity.

**Figure 7:**
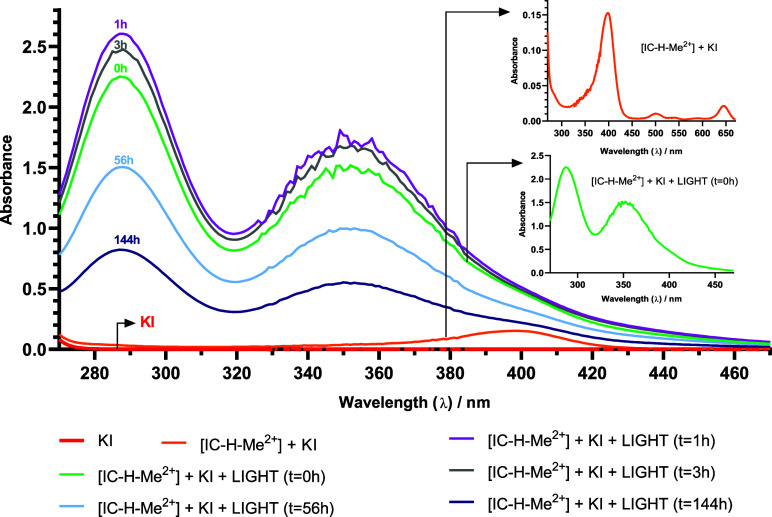
Absorption spectra of IC-H-Me^2+^ in PBS and in the presence of 50 mM KI, as a function of time for 6 days. KI alone has an absorption that is only noticeable at 270 nm. Spectra before and immediately after absorption are shown as insets.

I_3_^–^ has a characteristic absorption spectrum in water, with intense bands at 290 and 350 nm.^31^Figure 7 shows that the illumination of IC-H-Me^2+^ in the presence of KI rapidly gives the spectrum expected for I_3_^–^, which decays in the course of several days. Figure S3 shows that a similar spectrum and decay is obtained when I_2_ is added to KI. This is very compelling evidence that the species inactivating biofilms over the course of several hours after illumination is I_3_^–^. Definitive evidence was obtained adding I_2_ to I^–^_(aq)_ to obtain (i) a spectrum with an absorption intensity identical to that observed immediately after the illumination of IC-H-Me^2+^ in the presence of KI, Figure S3, (ii) a similar slow decay of the spectrum, Figure S3, (iii) the same long-lasting ability to inactivate biofilms as aPDT+KI, Figure 7. The molar absorption coefficient of I_3_^–^, ε_350_ = 25700 M^–1^ cm^–1^,^32^ together with the intensity of the absorption immediately after the illumination of 1 μM IC-H-Me^2+^ in the presence of 50 mM KI with 5 J cm^–2^, allows us to estimate [I_3_^–^]_0_ ≈ 70 μM. The generation of much more I_3_^–^ than the initial concentration of IC-H-Me^2+^ can be understood considering that the precursor of I_3_^–^ is singlet oxygen, because the generation of ^1^O_2_ by energy transfer from the triplet state of the photosensitizer regenerates the ground-state of the photosensitizer and prepares it for a new cycle of light absorption and energy transfer. O_2_ and I^–^ can be consumed in the process because they are in large excess. The mechanism proposed by Hamblin:
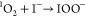
1
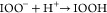
2
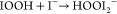
3
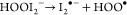
4with the disproportionations of the iodine radical anion^31^ and of the perhydroxyl radical,^33^ give the global reaction
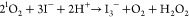
5which is in good agreement with our observations and those of Hamblin for aPDT with Photofrin, who showed that singlet oxygen is quenched by iodide, oxygen is consumed and hydrogen peroxide is generated.^20^ I_3_^–^ is in equilibrium with I_2_, which is the main cytotoxic species.

**Figure 8:**
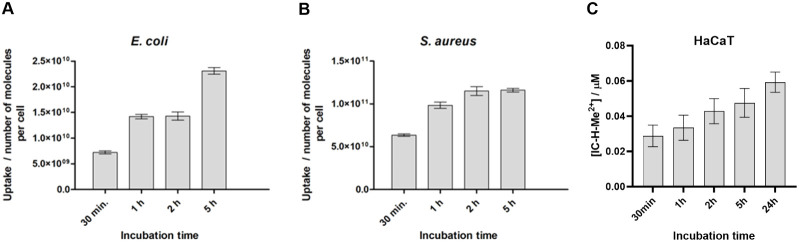
Cell uptake of 1 μM IC-H-Me^2+^, obtained from lysed cells in plate readers, based on fluorescence in the 600–700 nm range. (A) Planktonic *E. coli*. (B) Planktonic *S. aureus*. (C) HaCaT cells in monolayer.

Two important factors distinguish aPDT with IC-H-Me^2+^ combined with KI from trivial disinfection with antiseptics that contain I_2_, such as providone iodine^34^ or Lugol’s solution.^35^ The first factor is selectivity. Figure 5 shows that 1 h of incubation with 1 μM IC-H-Me^2+^ and 50 mM KI followed by 5 J cm^–2^, do not reduce mammalian cell viability to less than 70%. Nevertheless, Figure 4 shows that the same combination eradicates *S. aureus* and *E. coli* biofilms. This cannot be achieved with providone iodine or Lugol’s solution, which are cytotoxic to HeLa cells (cell viability lower than 70%) before reaching a concentration that is bactericidal (3 log CFU/ml reduction) to planktonic *E. coli*.^39^ I_2_ is always present in Lugol’s solution and starts reacting with cell components, namely proteins and unsaturated fatty acids,^37^ as soon as the solution is added to the cells. Attacking multiple biological targets is typical of antiseptics and has adverse effect on host cells, including fibroblasts and keratinocytes.^38^ Of note, we are comparing the eradication of biofilms with aPDT with the biocidal effect of antiseptics in planktonic bacteria, but planktonic bacteria are 100× easier to kill than biofilms. Selective eradication of biofilms is exceptional.

The second factor is the depth of the treatment. Figures 6 and 7 may give the impression that once I_3_^–^ is formed, it will stay active in the tissues for hours or days, eventually leading to cytotoxicity outside the infected tissues. The long lifetime of I_3_^–^ in PBS is misleading because iodine solutions lose their germicidal activity when they come into contact with organic load.^37^ It has been shown that providone iodine or Lugol’s solution are not toxic to mammalian or bacterial cells in the presence of culture medium,^36^ and we confirmed that this is also the case for I_2_+KI and for PDT with IC-H-Me^2+^ combined with KI (Figures S4 and S5). Indeed, Figure S6 shows that the absorption spectrum of I_3_^–^ is not observed in the I_2_+KI system when water is replaced by cell culture medium. Treatments with iodine-containing antiseptics are superficial because I_3_^–^ is rapidly consumed in reactions with a variety of biomolecules. In order to enhance penetration, large quantities must be employed at the cost of significant cytotoxicity. On the contrary, with aPDT it is possible to wait a reasonable amount of time (e.g., 1 h) to allow for photosensititzer and I^–^ infiltration in the biofilm, and then generate I_3_^–^*in situ*. This allows for treatments with a depth determined by the diffusion of the photosensitizer and by the penetration of light in tissues.

Selective eradication of biofilms with low cytotoxicity to host cells is unprecedented in aPDT using red light. We further investigated this selectivity evaluating the uptake of 1 μM IC-H-Me^2+^ by *E. coli*, *S. aureus* and HaCaT cells. Chlorin accumulation in planktonic bacteria was followed as a function of time, from 30 min to 5 h of incubation, for 1 μM solutions, based on the fluorescence of chlorin after their lyse with SDS 10% (Figure 8). IC-H-Me^2+^ accumulation reaches ∼1.2 × 10^11^ molecules per *S. aureus* cell in 2 h but it is only ∼2.5 × 10^10^ molecules per *E. coli* cell after 5 h. This is consistent with the difficulty of penetration of the cell wall of Gram-negative bacteria^4^ and with the higher concentrations required to photoinactivate *E. coli* presented in Figure 1. An independent flow cytometry study of uptake in planktonic *E. coli* showed stabilization of the uptake at short incubation times (Figure S7). Figure 8 also shows uptake by HaCaT cells increases over longer times. This is consistent with the higher phototoxicity of IC-H-Me^2+^ to HaCaT cells after 24 h of incubation (Figure S1).

**Figure 9:**
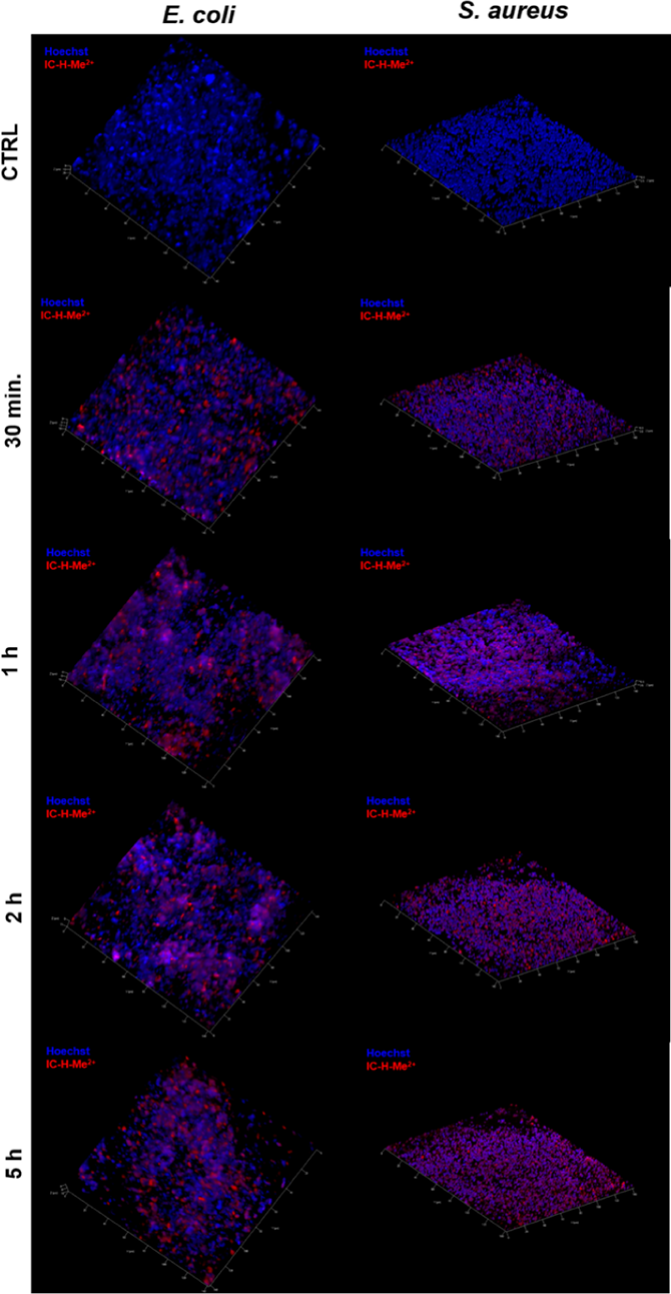
Representative confocal images of *E. coli* and *S. aureus* biofilms stained with Hoechst (blue) and after incubation with 1 μM IC-H-Me^2+^ for 30 min to 5 h. CTRL biofilms were not incubated with IC-H-Me^2+^.Blue fluorescence locates the biofilm and red fluorescence identifies the presence of IC-H-Me^2+^.

The infiltration of IC-H-Me^2+^ in biofilms can be conveniently followed by confocal fluorescence microscopy taking advantage of its characteristic fluorescence above 650 nm (red) when excited at 505 nm (green). The visualization of the biofilm was enabled by incubation with Hoechst 33342 solution, which stains DNA and emits at 461 nm (blue) when excited at 350 nm (UV). Figure 9 shows that before the addition of IC-H-Me^2+^ to the biofilms, only blue fluorescence can be detected and it informs on the presence of bacteria. The intensity of red fluorescence increases with the incubation time, as expected from increased infiltration of IC-H-Me^2+^ in the biofilm. Nevertheless, we see intense red fluorescence from the interior of the biofilm with 1 h of incubation, which means that IC-H-Me^2+^ was not blocked in its periphery. The selectivity of aPDT with IC-H-Me^2+^ can be related by its fast uptake by bacteria and by biofilms. We note that the bacteria were washed several times before collecting the fluorescence to ensure that the red fluorescence comes from the interior of the bacteria or of the biofilms.

Although confocal microscopy shows that IC-H-Me^2+^ infiltrates biofilms, it does not inform on the partition of IC-H-Me^2+^ between the extracellular matrix of the biofilm and the interior of the bacteria. In view of the dramatic potentiation of KI in the inactivation of biofilms (Figures 2 and 4), we hypothesize that IC-H-Me^2+^ is mostly in the extracellular matrix and that I_3_^–^ generated *in situ* has the ability to diffuse to the interior of the bacteria.

The remarkable phototoxicity of IC-H-Me^2+^ to bacteria at low concentrations, and its low cytotoxicity to HaCaT cells, encouraged the implementation of an exploratory study to investigate the feasibility of treating infected wounds with this photosensitizer. The model employed was that of abrasion wounds infected with bioluminescence bacteria,^14,30^ which in our case is *E. coli* expressing Green Fluorescent Protein (GFP). Three mice were employed as control (no chlorin and no light) and two groups of three mice each were treated (20 μM IC-H-Me^2+^ and 50 J cm^–2^ or 120 J cm^–2^ at 652 nm). This exploratory study did not include the combination with KI, which needs to be addressed with a proper formulation. The concentration of IC-H-Me^2+^ was increased to 20 μM based on the data in Figure 2. The light doses *in vivo* must be higher than *in vitro* because of the scatter and absorption of 650 nm light by skin. For example, taking 1.9 mm as the optical penetration depth of 650 nm light in skin, a surface dose of 50 J cm^–2^ is lowered to 6 J cm^–2^ at a 4 mm depth.

**Figure 10:**
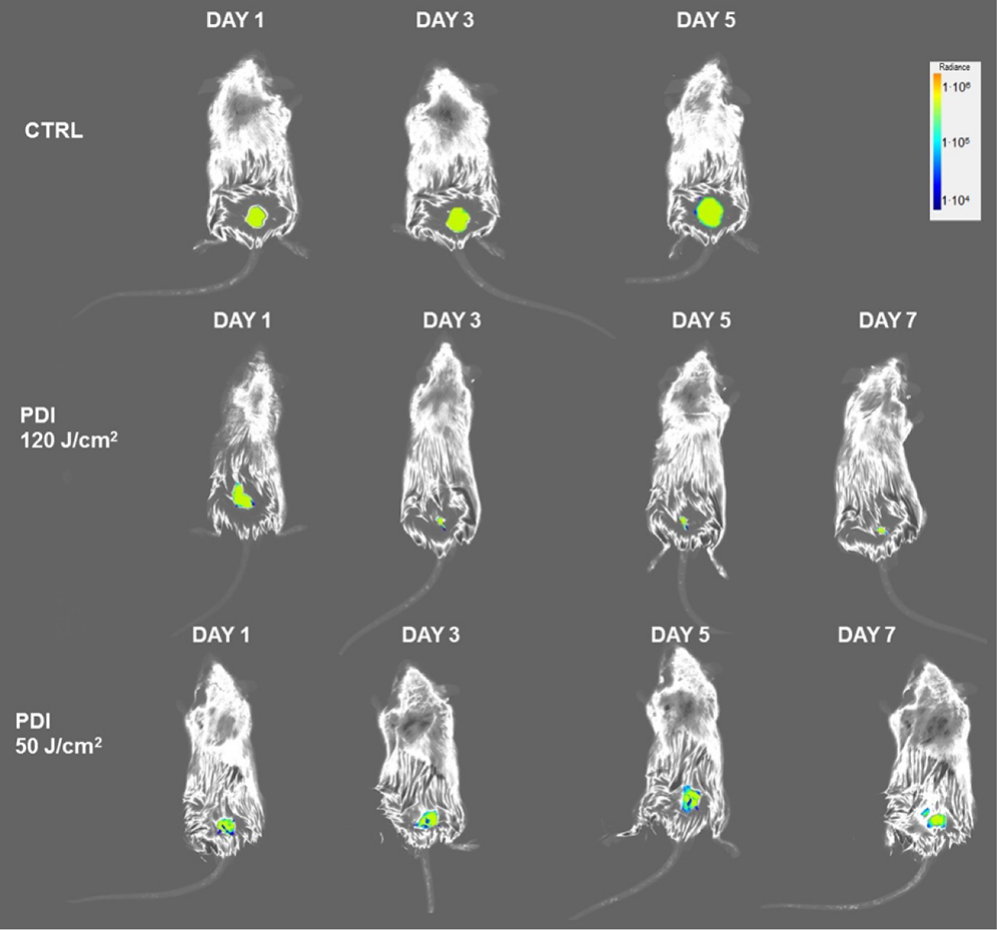
Fluorescence photographs of wounds in the back of mice infected with *E. coli* expressing GFP. Representative fluorescent images showing control and treatments with 20 μM IC-H-Me^2+^ and 50 J cm^–2^ or 120 J cm^–2^ at 652 nm.

The fluorescence intensity of GFP indicates the extension of the infection (Figure 10). The fluorescence in the control group progresses rapidly, in the group treated with 50 J cm^–2^ it seems to stabilize, and in the group treated with 120 J cm^–2^ it is much reduced by day 7. Compared with a similar infection treated with the corresponding porphyrin (25 μM IP-H-Me^2+^ and 120 J cm^–2^ at 420 nm),^14^ we remark a much faster reduction in the number of bacteria manifested by diminished fluorescence.

## Conclusions

IC-H-Me^2+^ exhibits the physical and photochemical properties of the “perfect” photosensitizer for aPDT.^15,16^ This chlorin fulfills the promise of eradicating planktonic *S. aureus* and *E. coli* at submicromolar concentrations with a light dose of 5 J cm^–2^ and 1 h of incubation, which are not significantly toxic to mammalian cells. The “therapeutic window” for planktonic bacteria is very wide, but narrows appreciably for biofilms. Nevertheless, it was possible to eradicate mature bacterial biofilms combining 1 μM IC-H-Me^2+^ with 50 mM KI and illuminating with 5 J cm^–2^ after 1 h of incubation. The potentiation of aPDT with KI is due to the *in situ* generation of I*_2_*. The selection of the incubation time is important to ensure high uptake by bacteria and biofilms while keeping mammalian cell uptake at moderate levels. Human cell viability remains above 70% under the conditions of biofilm eradication. *E. coli* colonization of wounds in the back of Balb/c mice were controlled with one single treatment employing 20 μM IC-H-Me^2+^ and 120 J cm^–2^ at 652 nm. Topical formulations of photosensitizers intended for local treatment of infected wounds through PDT have been classified as nonsterile medical devices, and in such cases the international standard establishes the threshold of 70% cell viability as the upper limit of tolerable cytotoxicity.^39^ Our results offer the basis for the development of formulations that selectively eradicate biofilms established in infected wounds using PDT.

## Materials and Methods

### Chemicals and Reactants

Potassium iodide (KI) and iodine (I_2_) were purchased from Sigma-Aldrich (Algés, Portugal) and used as received. The photosensitizer 5,15-bis(1,3-dimethylimidazol-2-yl)chlorin diiodide (IC-H-Me^2+^) was prepared as previously reported, and its HPLC purity shown to be >95%, as the average of the areas of the chromatograms with detection at 385 and 405 nm.^15^ IC-H-Me^2+^ stock solutions were prepared in phosphate-buffered saline (PBS) and stored at 4 °C in the dark. The stock IC-H-Me^2+^ solution was diluted in PBS to obtain the desired concentrations. The KI stock solution was prepared in PBS and diluted in the same solvent. I_2_ was dissolved in KI and diluted in PBS. Only fresh solutions of I_2_ were employed in the experiments.

### Bacteria Cells

Standard bacteria strains from American Type Culture Collection (ATCC) commonly used as controls for antibiotic susceptibility testing were employed. The bacteria strains employed were *E. coli* ATCC 25922, *S. aureus* ATCC 29213 and *E. coli* 243, which is a clinical strain resistant to ciprofloxacin.

### Photoinactivation of Planktonic Bacteria

The planktonic bacterial cells were cultured in Mueller-Hinton (MH) agar (Sigma-Aldrich) at 37 °C overnight. Cell density was adjusted to the 0.5 McFarland standard in sterile water, which corresponds to approximately 1.5 × 10^8^ CFU/mL. For aPDT experiments, bacteria suspensions in sterile water were added to 96-well plates and incubated in the dark for 60 min at room temperature with photosensitizer and, in other experiments, also with 50 mM KI. At the end of the incubation time, the plates were irradiated with a LED lamp from HIGROW LED (model GL36A), emission maximum at 660 nm with light dose (LD) of 5 J cm^–2^. The fluence of the LED was measured with a Coherent Laser Check power meter. The light dose absorbed by the compound was corrected by LED light emission overlap with compound absorption using the multiplicative factor of 0.6.^40^ Cells incubated with photosensitizers in the dark were covered with aluminum foil for the same time as the aPDT groups. After irradiation (or dark incubation), the samples were shaken, diluted in PBS, and mixed. Aliquots were taken from each well, streaked in MH agar in duplicate for CFU determination, and incubated at 37 °C for 24 h in the dark. After 24 h, the colonies were counted.

### Biofilm Growth

A colony of bacteria was suspended in Brain-Heart Infusion (BHI) broth and grown overnight in a shaker incubator (New Brunswick Scientific, Model G-25) at 120 rpm under aerobic conditions at 37 °C. An aliquot from an overnight bacterial suspension was refreshed in fresh BHI. Cell density was adjusted to the 0.5 McFarland standard in sterile water, which corresponds to approximately 1.5 × 10^8^ CFU/mL. Aliquots of the diluted bacterial suspensions were inoculated into 24-well U-bottom sterile polystyrene microplates and incubated for 24 h at 37 °C.

### Inactivation of Biofilms

The previously prepared plates with grown biofilms were incubated with the photosensitizer and, in some conditions, also 50 mM KI for 60 min in the dark at room temperature. Wells used as controls were incubated with PBS only. After the incubation period, the plate was irradiated with 660 nm LED with a LD of 5 J cm^–2^. Following irradiation, the biofilms were scraped carefully and sonicated. Treated and untreated samples were serially diluted, plated on the MH Petri dishes, and incubated for 24 h at 37 °C in the dark to allow colony formation. After this time, the colonies were counted, and CFUs were determined.

### Formation of the Triiodide Species (I_3_^–^)

One μM IC-H-Me^2+^ and 50 mM KI were added and the absorption spectra of the solution was registered after irradiation with of 5 J cm^–2^ at 660 nm. I_3_^–^ solutions were prepared dissolving I_2_ in a 500 mM KI stock solution followed by dilutions. The I_3_^–^ absorption spectrum overlapping with the absorption spectra of 1 μM IC-H-Me^2+^ and 50 mM KI after 5 J cm^–2^ illumination was obtained when the initial I_2_ and I^–^ concentrations were 0.144 and 1.14 mM, respectively. With this excess of I^–^, most I_2_ is complexed with I^–^. Absorption spectra were recorded in a Shimadzu UV-2100 spectrophotometer. The samples were measured at room temperature using standard quartz cuvettes with an optical path of 1 cm.

### Inactivation of Biofilms with I_2_ and KI

We assessed if I_3_^–^ formed from I_2_ and KI has the same inhibitory effect on biofilms as IC-H-Me^2+^ with KI and illumination, by preparing a solution of I_3_^–^ with approximately the same absorption spectrum as I_3_^–^ formed by aPDT with KI. The plates with biofilms were incubated with the solution of I_2_ and KI for 60 min at room temperature. Wells used as controls were incubated with PBS only. After that, the biofilms were scraped carefully and sonicated. Treated and untreated samples were serially diluted, plated on the MH Petri dishes, and incubated for 24 h at 37 °C in the dark to allow colony formation. After this time, the colonies were counted, and CFUs were determined.

### Human Cell Lines

Human epidermal keratinocytes cell lines (HaCaT), kindly provided by CNC UC (Coimbra, Portugal), were employed to assess the cytotoxicity in the dark and the phototoxicity of IC-H-Me^2+^ and the cytotoxicity of I_2_+KI solutions. Cells were grown in Roswell Park Memorial Institute (RPMI) 1640 medium, without phenol red, and supplemented with 10% (v/v) heat-inactivated fetal bovine serum (FBS), 10 mM HEPES, 10 mM sodium bicarbonate and 100 U/ml penicillin. Cells were maintained at exponential growth in a humidified incubator with 5% CO_2_ at 37 °C. Cells were detached using trypsin-EDTA solution (Sigma-Aldrich), counted, and seeded at the desired density in plates.

### Cytotoxicity and Phototoxicity in Human Cells

The toxicity toward human cell lines was evaluated *in vitro* using resazurin assay to estimate the viability of the cells after the appropriate treatment. HaCaT cells (20,000 cells/well) were seeded in 96-well plates and left to adhere overnight. After cell attachment, photosensitizer solutions in PBS and, in some conditions, KI at a specified concentration, were added to the cell cultures and incubated at 37 °C in the dark. Next, the cells were washed once with PBS, and then illuminated with 5 J cm^–2^ at 660 nm, or kept an equivalent time in the dark for control experiments. This was done in PBS and, in designated experiments, in the culture medium. After that, the cells were washed with fresh medium, and plates were returned to the incubator for 24 h. The resazurin assay was performed 24 h after irradiation. The cells were incubated with resazurin (0.01 mg/mL) and its metabolic product, resorufin, was measured with a microplate reader (Biotek Synergy HT) using 528/20 nm excitation and 590/35 nm emission filters. Cell viability of each condition was compared to the untreated cells, where the level of resazurin metabolization was assumed as 100% of viability.

### HaCaT Cells Uptake

One μM IC-H-Me^2+^ was incubated with HaCaT cells for selected time intervals (30 min, 1 h, 2 h, 5 h, 24 h) in the dark at room temperature. Unbound photosensitizer was removed by washing three times in PBS without Ca^2+^ or Mg^2+^. After the third wash the cells were lysed in 1% triton in DMSO (1:99), and scratched. The cellular uptake of the photosensitizer was evaluated by fluorescence using excitation at 420 nm and emission at 645 nm (BioTek Instruments). Calibration curves in the same solvent were used for the determination of the photosensitizer concentration.

### Uptake by Planktonic Bacteria

One μM IC-H-Me^2+^ was incubated with planktonic *E. coli* or *S. aureus* for selected time intervals (30 min, 1 h, 2 h, 5 h) in the dark at room temperature. Unbound photosensitizer was removed by washing twice in PBS without Ca^2+^ or Mg^2+^. After the second wash the cells were lysed in 10% SDS for 24 h. The cellular uptake of the photosensitizer was evaluated by fluorescence using excitation at 505 nm and emission between 600 and 700 nm (Tecan Infinite M200 Reader). Calibration curves were prepared in 10% SDS and used for the determination of photosensitizer concentration. Uptake values were obtained by dividing the photosensitizer concentration by the number of CFU. The number of molecules per cell was calculated from concentration. The cellular attachment/uptake of the chlorin was also evaluated with flow cytometry, quantifying it based on the intrinsic red fluorescence of IC-H-Me^2+^. To perform this analysis, bacteria cells were incubated with concentration 1 μM IC-H-Me^2+^ for selected time intervals in PBS. Subsequently, the cells underwent two washes with PBS and were prepared for analysis. The bacteria were then centrifuged and resuspended in 100 μL of PBS and examined using a Guava easyCyte flow cytometer. The acquired data were processed with InCyte software (MerckMillipore, Burlington, MA, USA) dedicated to this equipment.

### Uptake by Biofilms

To prepare biofilms, bacteria grown on suitable agar overnight, were suspended in a growth medium, and the optical density at 490 nm was adjusted to 0.65. The resulting bacterial suspension was then diluted 1:6, which involved mixing 1 mL of the bacterial suspension with 5 mL of prewarmed medium. The diluted suspension was then placed in an incubator at 37 °C with 5% CO_2_ for roughly 3 h to reach the mid logarithmic growth phase. Next, the mid log growth suspension was additionally thinned at a ratio of 1:2500 using prewarmed medium, and 200 μL of this diluted mixture was introduced into each well of an 8-well chamber slide coated with a thin layer of agar. After approximately 24 h, the medium from each chamber was aspirated and replaced with fresh medium. The biofilm was then exposed to 1 μM IC-H-Me^2+^ for selected time intervals and subsequently visualized using fluorescence microscopy. To visualize the biofilm after the incubation with the chlorin, the following steps were carried out: (i) the medium from each chamber was carefully removed, and the biofilm was washed twice gently with sterile saline; (ii) Hoechst solution (10 μg/mL) was added into each well and allowed to incubate at room temperature for 15 min while shielded from light; (iii) after the incubation, the staining solution was aspirated, and the biofilm was washed again with sterile saline, as performed previously; (iv) formalin (3.7% PFA) was introduced into each well, and the samples were left at room temperature for 30 min to fix the biofilms; (v) the biofilm was washed twice with saline, and the wash fluids containing formalin were discarded.; (vi) a mounting medium was applied, and a coverslip was placed on top of the sample; (vii), finally, the biofilms were visualized using a Zeiss880 confocal microscope, and the images obtained were subsequently analyzed using Zeiss ZEN software

### In Vivo Studies

The protocol of the pilot study was adapted from the publications of Hamblin and coworkers.^41^ Male Balb/c mice weighing 20–25 g, were shaved on the back the day before the experiment. Mice were anesthetized with an intraperitoneal injection of ketamine/xylazine cocktail for infection and imaging. The operative area of the skin was cleaned with alcohol, and a 25 mm^2^ square-shaped wound was created by scarification using a 27G needle. There was no visible bleeding within the wounds. Infection was carried out by applying 50 μL of a suspension of bacteria in PBS containing 2.5 × 10^7^ log-phase colony-forming units (CFU) of *E. coli*-GFP GFP (ATCC 25922). The 9 mice used in this pilot study were divided in one control group and two treatment groups. Imaging of the infection was carried out after a 15 min interval to allow the bacteria to bind to the wound tissue. IC-H-Me^2+^ was added 15 min after infection as 50 μL of 20 μM IC-H-Me^2+^ in PBS/DMSO (95/5). After additional 15 min to allow IC-H-Me^2+^ to bind to and infiltrate the bacteria the treated mice were then illuminated with 652 nm laser light (Omicron PDT- Laser, Model PDT652.1–2000, IEC60825, (1 – 2×) 2.0 ± 0.06 W, 90% of the emitted laser power are within ±4 nm centered around center operating wavelength). Mice in the two treatment groups were exposed to either 50 or 100 J cm^−2^ at 652 nm. Immediately after the end of PDT, the mice were resuscitated with an intraperitoneal injection of 0.5 mL sterile saline to prevent dehydration. All experiments were performed according to the 3R principles.

### Imaging

The Newton 7.0 Imaging System (Vilber) consists of an intensified change-coupled device camera mounted in a light-tight specimen chamber, fitted with a light-emitting diode, a setup that allowed for a background gray scale image of the entire mouse to be captured. In the photon-counting mode, an image of the emitted light from the bacteria was captured using an integration time of 2 min, at a maximum setting on the image-intensifier control module. Using the equipment software, the luminescence image was presented as a false-color image superimposed on top of the grayscale reference image. Before undergoing imaging, mice were anesthetized with intraperitoneal injections of 20 μL ketamine/xylazine (10:1) cocktail.

### Statistical Analysis

The experiments were performed in triplicate. The experiments were repeated at least three times. The statistical analysis was performed on GraphPad Prism 8. *In vitro* results are shown as Mean and Standard Error of the Mean. Statistical differences between populations were assessed with Student’s *t* test for unpaired data with unpaired variance: *, *p* ≤ 0.05; **, *p* ≤ 0.01; ***, *p* ≤ 0.001.

## Supporting Information Available

Additional data on HaCaT cell viability and on aPDT of *S. aureus* and *E. coli* in the presence of IC-H-Me^2+^ and KI, absorption spectra of IC-H-Me^2+^, KI and I_3_^–^, and flow cytometry of planktonic bacteria uptake (PDF)

## Abbreviations

AMR: antimicrobial resistance
aPDT: antimicrobial photodynamic therapy
*ATCC*: American Type Culture Collection
BCA: bicinchoninic acid
BHI: brain-heart infusion
CFU: colony-forming units
CNC UC: Centro de Neurociências da Universidade de Coimbra
CTR: control
DMSO: dimethyl sulfoxide
EDTA: ethylenediaminetetraacetic acid
DNA: DNA
EC_50_: half-maximum effective concentration
FBS: fetal bovine serum
GFP: green fluorescent protein
HEPES: 4-(2-hydroxyethyl)piperazine-1-ethanesulfonic acid
HPLC: high-performance liquid chromatography
IC-H-Me^2+^: 5,15-bis(1,3-dimethylimidazol-2-yl)chlorinate
IP-H-Me^2+^: 5,15-bis(1,3-dimethylimidazol-2-yl)porphyrinate
LED: light-emitting diode
MB: methylene blue
MH: Mueller Hinton
PBS: phosphate-buffered saline
PFA: paraformaldehyde
RPMI: Roswell Park Memorial Institute
SDS: sodium dodecyl sulfate
