# Supplementary material for: Efficient and Selective, In Vitro and In Vivo, Antimicrobial Photodynamic Therapy with a Dicationic Chlorin in Combination with KI
Source: ACS Infect Dis. 2024 Aug 16;10(9):3368–77. doi: 10.1021/acsinfecdis.4c00492 (PMC11406520; doi:10.1021/acsinfecdis.4c00492)
Supplement: Supplementary file 1 — id4c00492_si_001.pdf [file id4c00492_si_001.pdf]

SUPPORTING INFORMATION

Efficient and Selective, In Vitro and In Vivo, Antimicrobial Photodynamic Therapy with a Dicationic Chlorin in combination with KI

Anita S. Amorim,[†] Zoe A. Arnaut,[†] Ana I. Mata,[†] Barbara Pucelik,[‡] Agata Barzowska,[‡] Gabriela J. da Silva,[#] Mariette M. Pereira,[†] Janusz M. Dabrowski[‡] and Luis G. Arnaut[*,†]

Email: lgarnaut@ci.uc.pt

[†]CQC-IMS, Chemistry Department, University of Coimbra 3004-535 Coimbra, Portugal

[‡]Faculty of Chemistry, Jagiellonian University, 30-387 Kraków, Poland

[#]Faculty of Pharmacy of the University of Coimbra and Center for Neurosciences and Cell Biology, 3000-548 Coimbra, Portugal

Figure S1. Viability of immortalized human keratinocyte (HaCaT) cells after incubation with IC-H-Me^{2+}.

Figure S2. Cytotoxicity of IC-H-Me^{2+} to HaCaT cells in the dark, incubated for 24 h (A) or 1 h (B), in the presence of different KI concentrations.

Figure S3. Absorption spectra of IC-H-Me^{2+} with KI shortly after irradiation and of I_2 added to KI.

Figure S4. HaCaT cell viability after incubation with I_2+KI.

Figure S5. Survival of *S. aureus* biofilm in BHI broth after incubation with IC-H-Me^{2+}+KI.

Figure S6. Absorption spectra of DMEM, of I_2+KI in water and of I_2+KI in DMEM.

Figure S7. Flow cytometry of planktonic bacteria uptake of 1 μM IC-H-Me^{2+}.

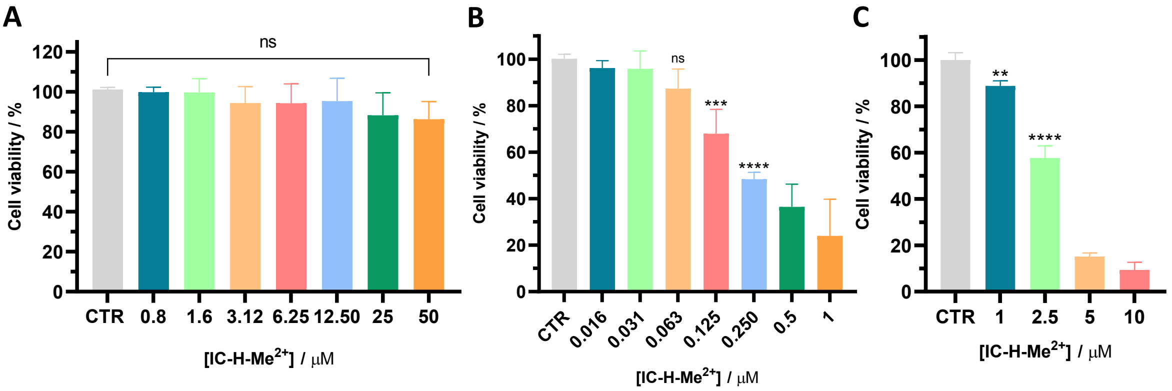

Figure S1. Viability of immortalized human keratinocyte (HaCaT) cells after incubation with IC-H-Me^{2+}. A) 24-hour incubation in the dark. B) 24-hour incubation light dose of 5 J cm^{-2}. C)
1-hour incubation and light dose of 5 J cm^{-2}. The symbols * ($p < 0.05$), ** ($p < 0.01$) and *** ($p < 0.001$) represent the statistical differences relative to control (CTR).

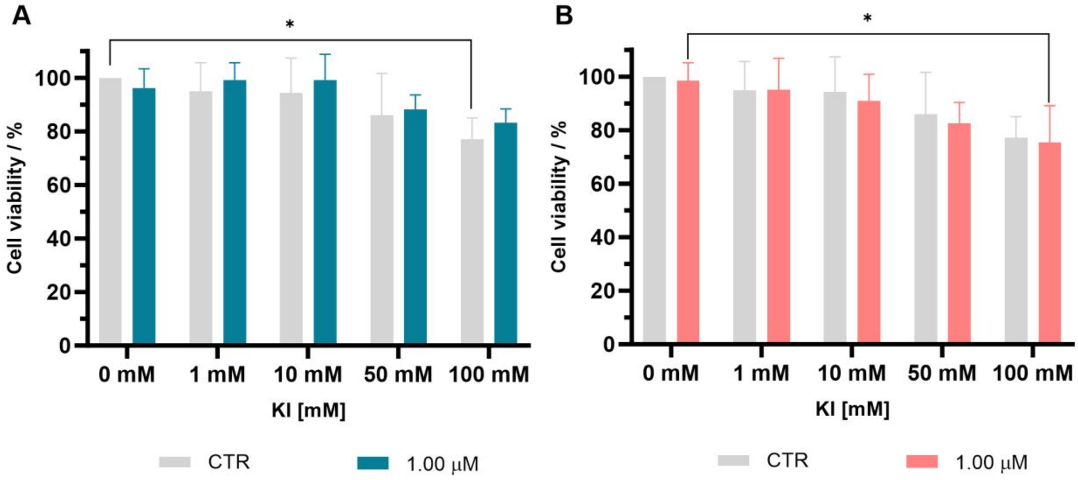

Figure S2. Cytotoxicity of 1 µM IC-H-Me^{2+} to HaCaT cells in the dark, incubated for 1 h (A) or 24 h (B), in the presence of different KI concentrations. Incubation with KI was always done for 1 hour. Results presented as mean ± SEM (N = 3). Statistic significant differences are identified as follows: *$p < 0.05$.

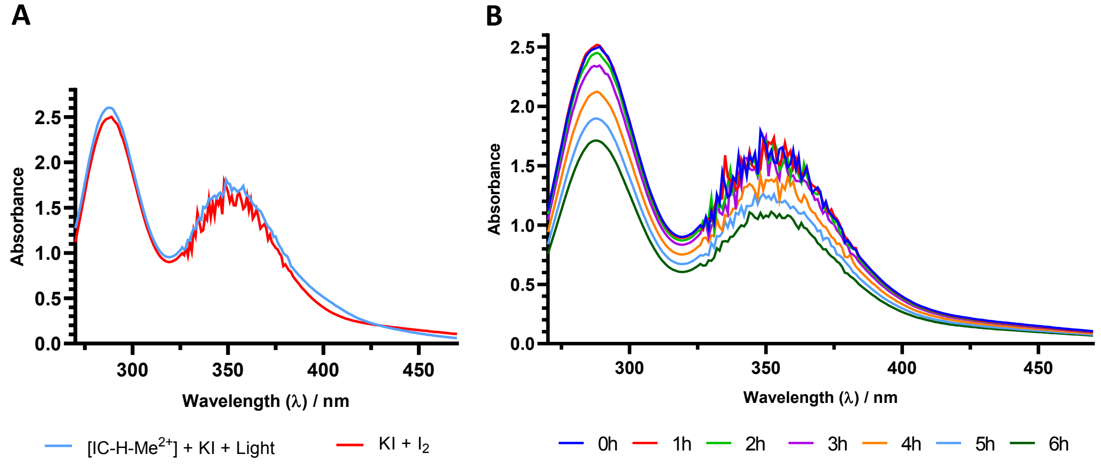

Figure S3. Absorption spectra of IC-H-Me^{2+} (1 μM) with KI (50 mM) shortly after irradiation with 5 J cm^{-2} and of I_2 (initially at 0.144 mM) added to KI (initially at 1.14 mM), and its decay over time.

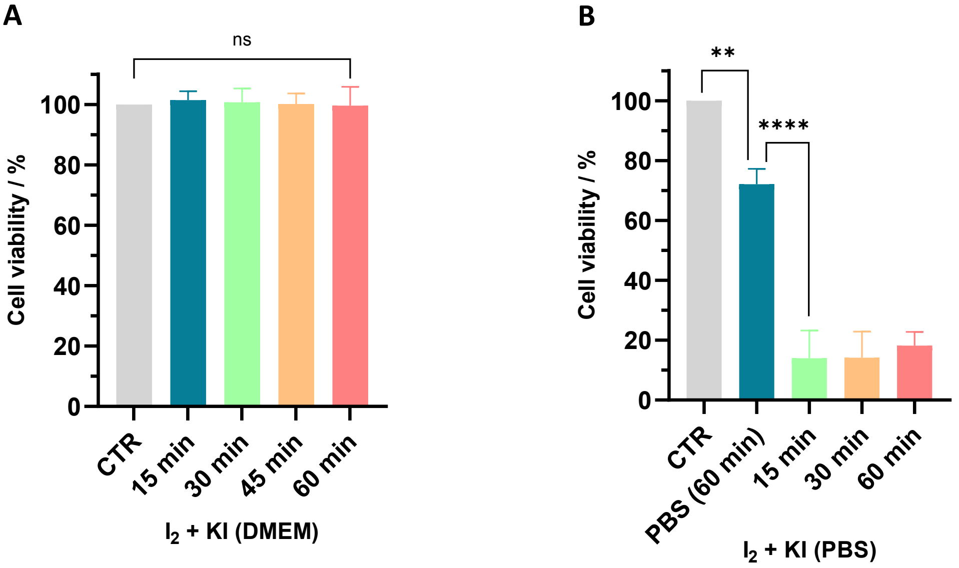

Figure S4. HaCaT cell viability after incubation with I_2+KI for the indicated times, relative to control (CTR) always in DMEM. A) in DMEM. B) In PBS, except CTR.

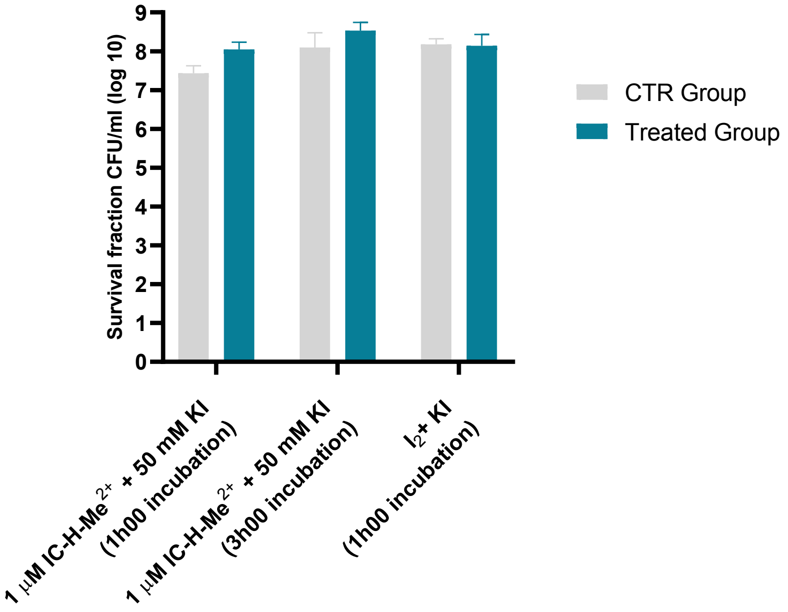

Figure S5. Survival of *S. aureus* biofilm in BHI broth after incubation with IC-H-Me^{2+}+KI for 1 h or 3h followed by illumination at 660 nm with 5 J cm^{-2}, or after incubation with I_2+KI for 1 h.

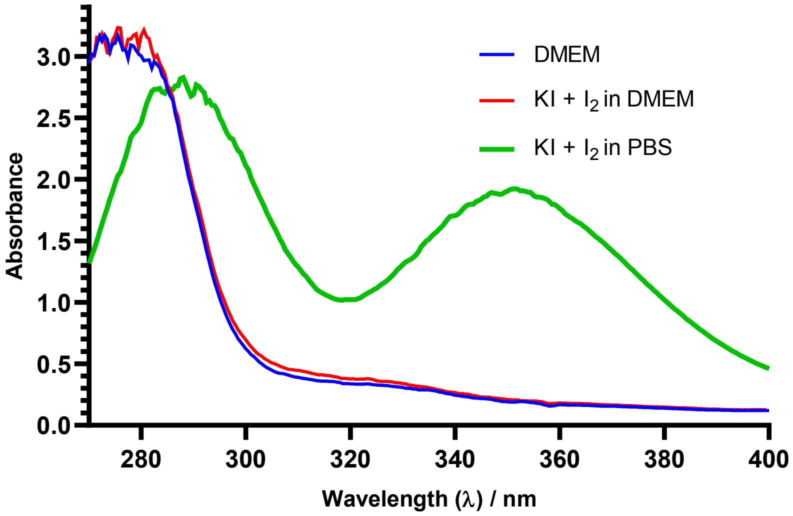

Figure S6. Absorption spectra of DMEM, of I_2+KI in water and of I_2+KI in DMEM, using initial concentrations of 0.144 mM for I_2 and 1.14 mM for KI.

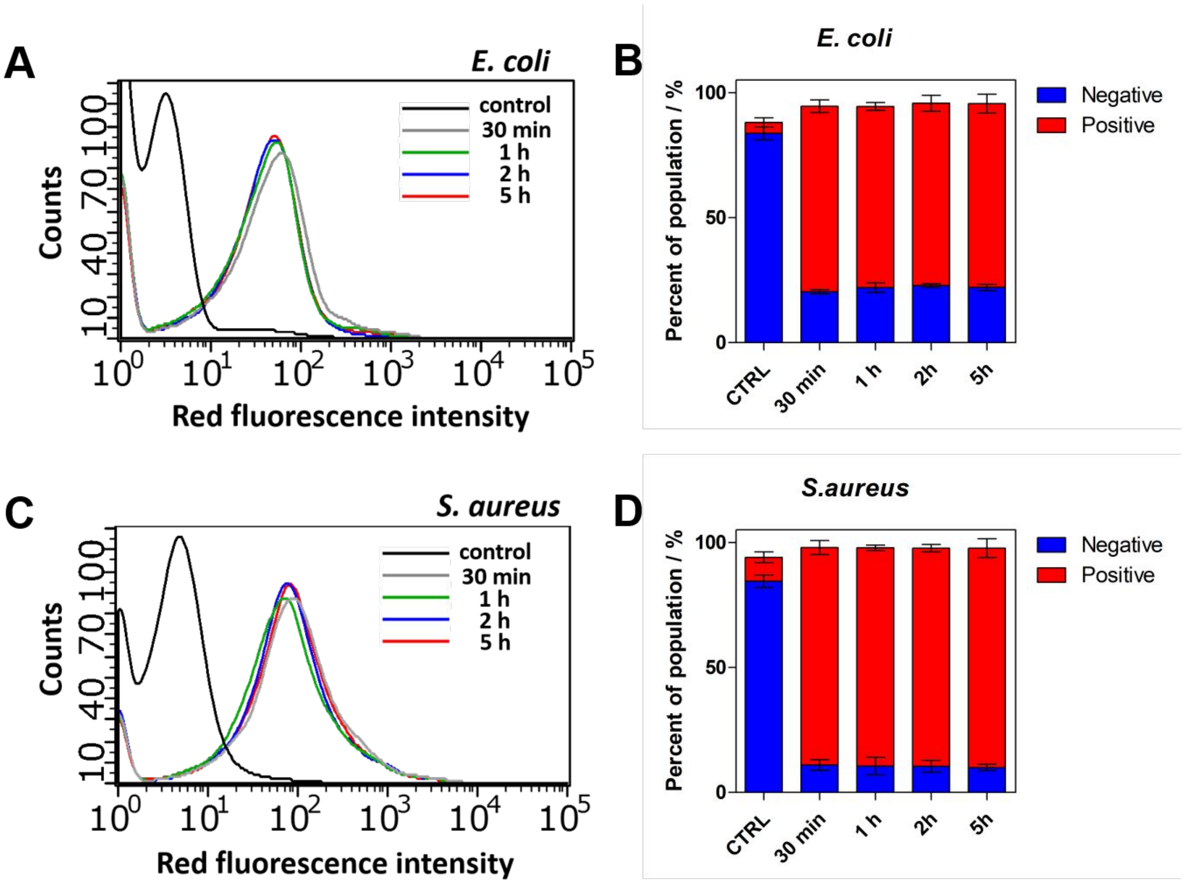

Figure S7. Flow cytometry of planktonic bacteria uptake of 1 μM IC-H-Me^{2+}.
